# Supplementary material for: Complexome profiling of the Chlamydomonas psb28 mutant reveals TEF5 as an early PSII assembly factor
Source: Plant Cell. 2025 Mar 17;37(6):koaf055. doi: 10.1093/plcell/koaf055 (PMC12164590; doi:10.1093/plcell/koaf055)
Supplement: koaf055_Supplementary_Data [file koaf055_supplementary_data.zip › psb28_interactive.zip!/plots/intensity/qualityPlots/peptides/_Cre09.g386756.t1.1.html]

### Cre09.g386756.t1.1

Trivial name:   
SeqCoverage [median]: 15.20  
Mass: 25.88 kDa  
  
 

| Sequence | MissedCleavage | Mass [Da] | wtIntensity | muIntensity | Score |
| --- | --- | --- | --- | --- | --- |
| GTTSPPGVTTDVIR | 0 | 1399.73 | 410.78 | 1307.64 | 95.48 |
| IDDVAKEPVLYAK | 1 | 1459.79 | 728.85 | 0.00 | 70.44 |
| VMLDQLPK | 0 | 942.52 | 0.00 | 500.67 | 75.74 |
|  |  |  | 1139.6 | 1808.3 |  |
